# Maternal and Fetal Lipid and Adipokine Profiles and Their Association with Obesity

**DOI:** 10.1155/2016/7015626

**Published:** 2016-04-12

**Authors:** Mario Solis-Paredes, Salvador Espino y Sosa, Guadalupe Estrada-Gutierrez, Sonia Nava-Salazar, Veronica Ortega-Castillo, Mario Rodriguez-Bosch, Eyerahi Bravo-Flores, Aurora Espejel-Nuñez, Maricruz Tolentino-Dolores, Rubí Gaona-Estudillo, Nancy Martinez-Bautista, Otilia Perichart-Perera

**Affiliations:** ^1^Department of Immunobiochemistry, Instituto Nacional de Perinatologia Isidro Espinosa de los Reyes, 11000 Ciudad de México, DF, Mexico; ^2^Posgrado en Ciencias Quimico-Biologicas, Escuela Nacional de Ciencias Biologicas, Instituto Politecnico Nacional, 11340 Ciudad de México, DF, Mexico; ^3^Direction of Education, Instituto Nacional de Medicina Genomica, 14610 Ciudad de México, DF, Mexico; ^4^Biomedical Research Branch, Instituto Nacional de Perinatologia Isidro Espinosa de los Reyes, 11000 Ciudad de México, DF, Mexico; ^5^Department of Obstetrics, Instituto Nacional de Perinatologia Isidro Espinosa de los Reyes, 11000 Ciudad de México, DF, Mexico; ^6^Department of Nutrition and Bioprogramming, Instituto Nacional de Perinatologia Isidro Espinosa de los Reyes, 11000 Ciudad de México, DF, Mexico; ^7^Immunology Department, Centro Medico Nacional La Raza, Instituto Mexicano del Seguro Social, 02990 Ciudad de México, DF, Mexico

## Abstract

*Background*. Maternal metabolic changes impact fetal metabolism resulting in a higher risk for developing chronic diseases later in life. The aim of this study was to assess the association between maternal and fetal adipokine and lipid profiles, as well as the influence of maternal weight on this association.* Methods*. Healthy pregnant women at term who delivered by C-section were enrolled. Maternal and fetal glucose, lipid profile, adiponectin, leptin, and resistin levels were analyzed by obesity and maternal weight gain. Statistics included descriptives, correlations, and mean differences (SPSS v20.0).* Results*. Adiponectin and resistin concentrations were higher in fetal blood, while leptin was lower (*p* < 0.05). A significant inverse association between maternal resistin and fetal LDL-cholesterol (LDL-C) (*r* = −0.327; *p* = 0.022) was observed. A positive correlation was found between maternal and fetal resistin (*r* = 0.358; *p* = 0.013). Women with excessive weight gain had higher leptin levels and their fetuses showed higher LDL-C levels (*p* < 0.05).* Conclusions*. Maternal resistin showed an inverse association with fetal LDL-C, suggesting that maternal adiposity status may play an active role in the regulation of fetal lipid profile and consequently, in fetal programming. Excessive maternal weight gain during pregnancy may exert an effect over metabolic mediators in both mother and newborn.

## 1. Introduction

The prevalence of overweight and obesity has risen dramatically worldwide and it is considered a severe public health problem [1]. In Mexico, 73% of women in reproductive age are overweight or obese [2], and during pregnancy both conditions have negative effects on several cellular and molecular mechanisms [3, 4]. Systemic inflammation caused by excessive adipose tissue in pregnant women superimposes an additional risk of developing preeclampsia, insulin resistance, gestational diabetes, and progression to type 2 diabetes mellitus (T2DM) [5].

Adipose tissue is a specialized endocrine and paracrine organ that produces different adipocytokines such as adiponectin, leptin, and resistin [6]. These molecules are involved in regulating appetite and energy balance, angiogenesis, inflammation, immunity, blood pressure, insulin sensitivity, glucose homeostasis, nutrient transport, and lipid metabolism [7].

Previous reports show that resistin, leptin, and adiponectin modulate serum lipids and cholesterol profile in general population. In adults, serum adiponectin concentration correlates positively with high-density lipoprotein cholesterol (HDL-C), whereas triglycerides (TG) are negatively correlated [8]. On the other hand, resistin has shown an inverse association with total cholesterol (TC), including HDL-C, low-density lipoprotein cholesterol (LDL-C), and adiposity [9]. Despite the fact that leptin concentration has not been associated with lipid profile, this adipokine is involved in weight gain and adiposity in adults, pregnant women, and newborns [10]. Altered levels of these adipokines are associated with chronic inflammation, insulin resistance, obesity, and cardiovascular disease [11].

It is well known that alterations in the metabolic status during pregnancy result in fetal programing for many metabolic diseases. However, it is not clear if maternal serum adipokines modulate fetal adipokine and lipid profiles. In this work we reported the association between maternal and fetal adipokine and lipid profiles, as well as the influence of maternal weight on this association, as potential factors implicated in fetal programming.

## 2. Subjects and Methods

### 2.1. Research Design and Study Population

This cross-sectional study was done during 2013-2014 at the* Instituto Nacional de Perinatologia* in Mexico City and is a secondary analysis from a larger study. The project was approved by the IRB (number 212250-22741); participation was voluntary and all participants signed the informed consent.

Sixty-seven healthy pregnant women at term (37–40 weeks of gestation by last menstrual period and obstetric ultrasonography), who delivered by elective or iterative cesarean section (C-section), were enrolled according to the inclusion criteria of the larger study. For this analysis, the following inclusion criteria were used: having age > 18 years, having pregestational body mass index (BMI) > 18.5 kg/m^2^, having singleton pregnancy, and having known pregestational weight. Exclusion criteria included multiple gestation, T2DM or gestational diabetes mellitus (GDM), chronic or gestational hypertension, renal or autoimmune disease, intrauterine fetal growth restriction, fetal structural abnormalities or drug intake that affects metabolism, and inflammation (metformin, steroids, insulin, and antihypertensives, among others).

### 2.2. Biochemical and Anthropometric Parameters

Maternal weight (digital scale Tanita BMB-800) and stature (cm) (SECA 220) were measured by trained personnel. Pregestational weight was self-reported, and pregestational BMI was calculated. Obesity classification was done with the World Health Organization criteria. Weight gain was categorized as adequate, low, or excessive at the end of pregnancy, according to Institute of Medicine (IOM) classification (2009) [12].

Maternal blood was obtained before C-section and fetal blood was collected from the umbilical cord vein after birth. Blood samples were centrifuged and serum was stored at −80°C until the assays were performed.

Seric TC, HDL-C, TG, and glucose were measured by enzymatic colorimetric methods using an automated analyzer (ISE Echo Lory 2000) and commercial kits (DiaSys Diagnostic Systems GmbH, Germany); LDL-C was calculated by the Friedewald equation [13]. Adiponectin, leptin, and resistin were quantified by enzyme-linked immunosorbent assay (ELISA) using commercially available kits according to the manufacturer's instructions (R&D Systems Inc., Minneapolis, MN, USA). Homeostatic model assessment (HOMA) index was calculated using fasting glucose and insulin determinations as previously described [14].

Newborn anthropometric assessment was performed within 24 to 72 hours after birth by a trained dietitian. Weight (digital scale, Tanita Baby & Mommy 1582) and length (portable stadiometer, SECA 207) were measured.

### 2.3. Statistical Analysis

Descriptive statistics and frequencies were used to describe the study population characteristics. Spearman's correlations were performed to study the association between maternal and umbilical cord blood lipids and adipokines. One-way ANOVA was used to analyze differences by pregestational BMI and weight gain categories, using the IBM SPSS v20.0 software. Data are expressed as mean ± SD, and *p* values ≤ 0.05 were considered statistically significant.

## 3. Results

Based on pregestational BMI, 29.4% (*n* = 20) of enrolled women had normal weight, 36.8% were overweight (*n* = 25), and 32.4% were obese (*n* = 22). At the beginning of the study, women were 32.3 ± 7.16 years old. More than half of the women were multiparous (61.8%).

Maternal anthropometric and biochemical parameters are summarized in Table 1. Women classified as overweight or obese were older than normal weight women. Total gestational weight gain and LDL-C concentrations were lower in the obesity group. There were no significant differences in adiponectin, leptin, resistin, fasting glucose, TG, TC, and HDL-C concentration by weight status.

Newborn anthropometric measurements as well as fetal lipid and adipokine profiles classified according to pregestational BMI and maternal weight gain are described in Tables 2 and 3, respectively. There were no significant differences in newborn anthropometric and biochemical parameters between the study groups (Table 2). Women with excessive weight gain had higher leptin levels when compared to low and normal weight gain groups; higher levels of LDL-C were observed in fetuses from these mothers (Table 3).

Adiponectin and resistin concentrations were higher in fetal blood compared to maternal blood (28 ± 15.1 versus 7.3 ± 4.8 *μ*g/mL; *p* < 0.001 and 20.8 ± 11.3 versus 14.5 ± 7.8 ng/mL; *p* < 0.001, resp.). On the contrary, leptin was lower in fetal than maternal blood (5.6 ± 4.8 versus 27.3 ± 36.5 ng/mL; *p* < 0.001).

**Figure 1:**
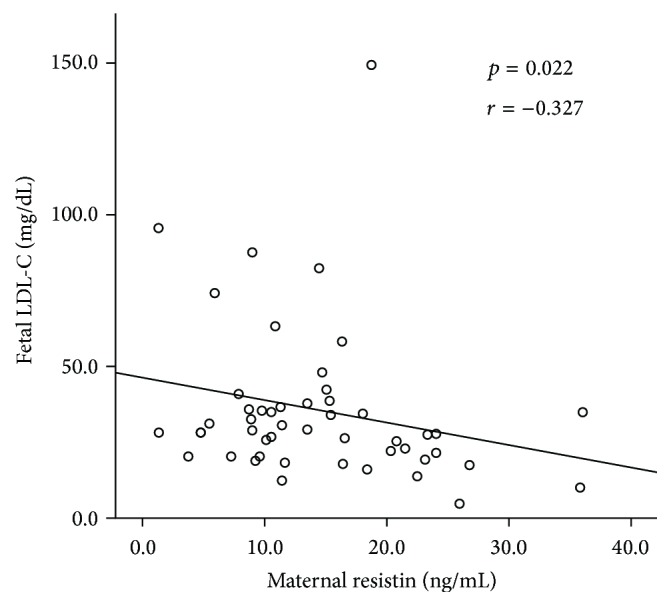
Correlation between circulating levels of maternal resistin and fetal LDL-C.

A significant inverse association between maternal resistin and fetal LDL-C (*r* = −0.327; *p* = 0.022) was observed (Figure 1). In addition, a positive significant correlation was found between maternal resistin and fetal resistin (*r* = 0.358; *p* = 0.013). Maternal serum leptin correlated significantly with maternal TC (*r* = 0.293; *p* = 0.048) and HDL-C (*r* = 0.369; *p* = 0.012). Maternal adiponectin was inversely correlated to fetal adiponectin; however this difference was not statistically significant (*r* = −0.300; *p* = 0.072) (Table 4).

## 4. Discussion

Altered adipokine levels in obesity and pregnancy may affect different metabolic pathways. The main finding of this study is the inverse association observed between maternal resistin and fetal levels of LDL-C; to the best of our knowledge, it has never been described before. Recently, it was demonstrated that resistin participates in the metabolic regulation of LDL-C. Placenta transports cholesterol from maternal circulation to the fetus by cholesterol transporters that include low-density lipoprotein receptor (LDLR), the very low-density lipoprotein receptor (VLDLR), and the SRBI receptor [15]. A previous study found that, in primary hepatocyte cultures as well as in HepG2 cells, elevated resistin levels inhibit the expression of LDLR. The proposed mechanism for this action is that resistin induces the expression of the PCSK9 protein, which regulates the degradation of LDLR, by joining its extracellular domain at the cellular surface [16, 17]. Studies in human hepatocytes have also shown that resistin levels ≥25 ng/mL inhibit LDLR expression [17]. In our study, LDL-C levels were lower in fetuses from women with higher resistin levels (≥20.1 ng/mL) when compared to fetuses from women with lower resistin levels (≤20 ng/mL). These results suggest that, in women with high resistin levels, LDL-C transport and efflux mechanisms to the fetus may be altered, possibly by inhibition of placental LDLR. Endogenous resistin production by the placenta has been proposed by some authors [18], and a weak association has been reported between placental resistin expression and circulating resistin [19]. Therefore, more studies need to be conducted to investigate the effect of placental resistin on placental LDLR.

In our study, fetal adiponectin and resistin levels were much higher than maternal concentrations, and fetal leptin was lower than in the mothers. These higher levels of fetal adipokines could be explained by their role as endocrine signals for placental development and fetal growth. In addition, adipokines participate in gluconeogenesis regulation and energy balance in later life [20, 21].

In this study, a positive correlation between maternal leptin levels with maternal TC and HDL-C was observed. According to this, some studies published before have reported an association between maternal leptin and HDL-C [22, 23], but other studies have failed to show this association [24, 25]. In women with obesity and diabetes, maternal leptin is positively associated with HDL-C but negatively associated with TG [26]. Therefore, altered leptin levels during pregnancy appeared to be related not only to adiposity, but also to lipid alterations.

When maternal lipid or adipokine levels were analyzed by obesity presence or by maternal weight gain, no differences were observed, except for higher leptin levels in women classified with excessive weight gain at the end of pregnancy, when compared to women who had adequate or low weight gain. Leptin is a hormone that regulates energy homeostasis and body weight [27], and excessive weight gain is related to excessive fat accumulation [28]. Other studies have reported association between high maternal leptin with maternal obesity and excessive weight gain [29]. Recent studies in obesity during pregnancy show that resistin appeared to be higher in obese women [30, 31] and in women with higher visceral adiposity [32], however, other studies have failed to show this association [33].

It has been described that excessive maternal adiposity may influence placental functions, specifically those related to the transportation and metabolism of lipids, resulting in altered fetal lipid profiles [34]. This is consistent with our finding that LDL-C levels were higher in fetuses from women with excessive weight gain.

No other differences were observed in lipids of fetuses from women who were classified as being normal weight, overweight, or obese before pregnancy. Lemas et al. found an inverse association between maternal BMI and fetal HDL-C [35], but very few studies exist. It should be noted that the fetal lipid profile may also be influenced by individual maternal lipid profiles, not only by the presence of obesity [36, 37].

As reported before [21], fetal resistin was not associated with birth weight in this study. It has been described that macrosomic newborns (>4000 g) have higher adiponectin levels [38] and that cord blood leptin levels show a moderate correlation with birth weight and maternal BMI [35, 39].

This study has some limitations that should be acknowledged: (a) the small sample may decrease the power of associations, (b) only women with C-section were included in the study, lowering its external validity, and (c) the studied variables are affected by many maternal and fetal factors that were not taken into account. In future studies, adjusting for confounding factors such as diet, baseline lipid profile, age, and parity should be guaranteed.

## 5. Conclusion

This study shows that excessive maternal weight gain is associated with higher maternal leptin levels and higher fetal LDL-C. Likewise, maternal resistin showed an inverse association with fetal LDL-C, suggesting that maternal adiposity status may play an active role in the regulation of fetal lipid profile and consequently in fetal programming. Ongoing work in our lab is conducted to study whether resistin modulates lipid receptors in the placenta, specifically LDLR, to elucidate the molecular mechanism of resistin and LDL-C association.

## Figures and Tables

**Table 1:** Maternal anthropometric and biochemical measurements according to the pregestational BMI category.

Variables	Pregestational BMI category (kg/m^2^)		
	Normal 18.5 to <25.0 (*n* = 20)	Overweight 25.0 to <30.0 (*n* = 25)	Obese ≤30.0 (*n* = 22)
Age (years)	27.7 ± 7.2	32.3 ± 6.8^a^	35.6 ± 5.5^b^
Pregestational BMI (kg/m^2^)	22.6 ± 1.6	27.3 ± 1.4^a^	34.5 ± 3.5^b,c^
Total gestational weight gain (kg)	12.4 ± 4.9	11.0 ± 5.7	5.7 ± 6.0^b,c^
Adiponectin (*μ*g/mL)	9.5 ± 5.8	5.7 ± 3.7	6.9 ± 4.3
Leptin (ng/mL)	17.5 ± 14.9	30.9 ± 23.5	32.6 ± 55.5
Resistin (ng/mL)	14.1 ± 6.0	14.4 ± 8.4	15.0 ± 8.9
Fasting glucose (mg/dL)	84.1 ± 27.2	86.5 ± 25.0	89.2 ± 22.5
TG (mg/dL)	233.9 ± 95.3	221.5 ± 50.8	221.4 ± 93.1
TC (mg/dL)	237 ± 75.0	213.8 ± 57.3	187.7 ± 63.1
HDL-C (mg/dL)	63.1 ± 17.0	61.6 ± 13.9	54.9 ± 13.7
LDL-C (mg/dL)	121.6 ± 43.8	107.9 ± 43.9	84.7 ± 41.2^b^

BMI: body mass index; TG: triglycerides; TC: total cholesterol; HDL-C: high-density lipoprotein cholesterol; LDL-C: low-density lipoprotein cholesterol. Values represent mean ± SD. *p* values were estimated using one-way ANOVA. ^a^ *p* < 0.05 overweight versus normal; ^b^ *p* < 0.05 obese versus normal; ^c^ *p* < 0.05 obese versus overweight.

**Table 2:** Newborn anthropometry and fetal biochemical parameters according to pregestational BMI category.

Variables	Pregestational BMI category (kg/m^2^)		
	Normal 18.5 to <25.0 (*n* = 20)	Overweight 25.0 to <30.0 (*n* = 25)	Obese ≤30.0 (*n* = 22)
Gestational age at birth (weeks)	38.8 ± 1.8	38.5 ± 1.4	38.4 ± 0.9
Newborn weight (g)	2789.3 ± 697.3	2941.3 ± 435.1	3098.5 ± 307.5
Adiponectin (*μ*g/mL)	26.5 ± 16.3	28.7 ± 14.2	28.5 ± 15.7
Leptin (ng/mL)	4.9 ± 3.4	5.3 ± 4.8	6.4 ± 5.9
Resistin (ng/mL)	20.3 ± 13.9	18.3 ± 5.6	23.5 ± 12.7
Fasting glucose (mg/dL)	60.9 ± 18.9	63.3 ± 13.5	62.0 ± 19.6
TG (mg/dL)	64.0 ± 85.3	34.8 ± 40.1	36.3 ± 27.9
TC (mg/dL)	85.2 ± 44.3	69.7 ± 42.3	66.3 ± 19.5
HDL-C (mg/dL)	38.0 ± 12.1	31.8 ± 9.3	31.4 ± 9.8
LDL-C (mg/dL)	35.0 ± 21.2	30.9 ± 27.8	30.5 ± 21.2

TG: triglycerides; TC: total cholesterol; HDL-C: high-density lipoprotein cholesterol; LDL-C: low-density lipoprotein cholesterol. Values represent mean ± SD. There were no significant differences between the study groups.

**Table 3:** Maternal and fetal adipokine and lipid profiles according to maternal weight gain status.

Variables	Maternal weight gain category					
	*N*	Low	*N*	Normal	*N*	Excessive
Adiponectin_M_ (*μ*g/mL)	16	7.2 ± 4.9	12	8.5 ± 5.4	12	6.2 ± 4.1
Adiponectin_F_ (*μ*g/mL)	24	26.8 ± 10.6	20	31.5 ± 16.8	16	25.5 ± 18.5
Leptin_M_ (ng/mL)	21	15.4 ± 13.4	13	21.8 ± 27.2	14	50.3 ± 5.5^a,b^
Leptin_F_ (ng/mL)	25	4.2 ± 3.1	20	6.81 ± 5.3	17	6.1 ± 6.0
Resistin_M_ (ng/mL)	21	14.4 ± 7.0	14	15.5 ± 9.0	14	13.7 ± 8.0
Resistin_F_ (ng/mL)	26	21.6 ± 11.8	19	19.7 ± 11.1	16	20.7 ± 11.2
Fasting glucose_M_ (mg/dL)	21	86.9 ± 25.4	13	94.6 ± 29.7	13	78.9 ± 13.3
Fasting glucose_F_ (mg/dL)	26	63.6 ± 18.1	21	60.7 ± 15.3	17	61.5 ± 18.6
TG_M_ (mg/dL)	21	214.1 ± 85.9	13	213.5 ± 55.1	13	254.8 ± 92.8
TG_F_ (mg/dL)	26	45.9 ± 46.0	21	28.3 ± 16.7	17	60.5 ± 88.5
TC_M_ (mg/dL)	21	211.2 ± 77.1	13	196.8 ± 52.0	13	223.8 ± 64.6
TC_F_ (mg/dL)	26	78.3 ± 32.1	21	59.7 ± 17.0	17	82.1 ± 55.6
HDL-C_M_ (mg/dL)	21	59.0 ± 14.2	13	55.3 ± 14.6	13	64.4 ± 16.1
HDL-C_F_ (mg/dL)	25	34.9 ± 10.6	21	31.5 ± 9.8	16	34.3 ± 12.0
LDL-C_M_ (mg/dL)	20	102.7 ± 50.2	13	98.8 ± 36.5	11	107.2 ± 46.0
LDL-C_F_ (mg/dL)	26	35.9 ± 21.3	13	22.5 ± 9.0	17	37.8 ± 34.3^a^

M: maternal; F: fetal; TG: triglycerides; TC: total cholesterol; HDL-C: high-density lipoprotein cholesterol; LDL-C: low-density lipoprotein cholesterol. Values represent mean ± SD. *p* values were estimated using one-way ANOVA. ^a^ *p* < 0.05 excessive versus normal; ^b^ *p* < 0.05 excessive versus low.

**Table 4:** Spearman's correlation coefficients between maternal and fetal adipokines.

Variable	Adiponectin_M_	Leptin_M_	Resistin_M_
Adiponectin_F_ (*μ*g/mL)	*r* = −0.300; *p* = 0.072	*r* = −0.040; *p* = 0.793	*r* = 0.133; *p* = 0.378
Leptin_F_ (ng/mL)	*r* = 0.058; *p* = 0.728	*r* = 0.133; *p* = 0.374	*r* = −0.180; *p* = 0.220
Resistin_F_ (ng/mL)	*r* = 0.038; *p* = 0.823	*r* = 0.063; *p* = 0.677	**r** = 0.358; **p** = 0.013

M: maternal; F: fetal. Values in bold type are statistically significant: *p* < 0.05.
